# Preventing and Controlling Zinc Deficiency Across the Life Course: A Call to Action

**DOI:** 10.1016/j.advnut.2024.100181

**Published:** 2024-01-26

**Authors:** Nicola M Lowe, Andrew G Hall, Martin R Broadley, Jennifer Foley, Erick Boy, Zulfiqar A Bhutta

**Affiliations:** 1 Center for Global Development, University of Central Lancashire, Preston, United Kingdom; 2 Department of Nutrition, University of California, Davis, CA, United States; 3 Department of Nutritional Sciences & Toxicology, University of California, Berkeley, CA, United States; 4 Rothamsted Research, West Common, Harpenden, United Kingdom; 5 School of Biosciences, University of Nottingham, Loughborough, United Kingdom; 6 HarvestPlus, International Food Policy Research Institute, Washington, DC, United States; 7 Center for Global Child Health, The Hospital for Sick Children, Toronto, ON, Canada; 8 Center of Excellence in Women and Child Health, Aga Khan University, Karachi, Pakistan

**Keywords:** zinc, zinc deficiency, biofortification, life course, evidence-based nutrition interventions

## Abstract

Through diverse roles, zinc determines a greater number of critical life functions than any other single micronutrient. Beyond the well-recognized importance of zinc for child growth and resistance to infections, zinc has numerous specific roles covering the regulation of glucose metabolism, and growing evidence links zinc deficiency with increased risk of diabetes and cardiometabolic disorders. Zinc nutriture is, thus, vitally important to health across the life course. Zinc deficiency is also one of the most common forms of micronutrient malnutrition globally. A clearer estimate of the burden of health disparity attributable to zinc deficiency in adulthood and later life emerges when accounting for its contribution to global elevated fasting blood glucose and related noncommunicable diseases (NCDs). Yet progress attenuating its prevalence has been limited due, in part, to the lack of sensitive and specific methods to assess human zinc status. This narrative review covers recent developments in our understanding of zinc’s role in health, the impact of the changing climate and global context on zinc intake, novel functional biomarkers showing promise for monitoring population-level interventions, and solutions for improving population zinc intake. It aims to spur on implementation of evidence-based interventions for preventing and controlling zinc deficiency across the life course. Increasing zinc intake and combating global zinc deficiency requires context-specific strategies and a combination of complementary, evidence-based interventions, including supplementation, food fortification, and food and agricultural solutions such as biofortification, alongside efforts to improve zinc bioavailability. Enhancing dietary zinc content and bioavailability through zinc biofortification is an inclusive nutrition solution that can benefit the most vulnerable individuals and populations affected by inadequate diets to the greatest extent.

**Statements of significance**

This review is the first to advance a broader perspective on the importance of zinc deficiency, from its well-known roles in child growth and infections to being a key player in the emergent global burden of diabetes and cardiovascular disease in adulthood. In light of recent research supporting an expanded recognition of the relevance of zinc deficiency across the life course and in the context of a rapidly changing climate, future directions for its identification and options for its control are discussed.

## Introduction

Zinc determines a greater number of critical life functions than any other single micronutrient. It has catalytic, structural, and regulatory roles that are essential to metabolic pathways, gene expression, hormone function, immune defense mechanisms, and much more [1]—making it vitally important to health and growth across the life course [2,3].

Zinc deficiency is one of the most common forms of micronutrient malnutrition globally, and yet remains among the least well recognized. An estimated 17% of the global population is at risk of inadequate zinc intake, with the prevalence as high as 19% and 24% in Asia and Africa, respectively [4,5].

The manifestations of zinc deficiency are nonspecific, and severity varies by age, duration, and the presence of other underlying diseases [6]. In children in low- and middle-income countries (LMICs), it is a significant limiting factor for growth [7], contributing to stunting in apparently healthy [8] as well as malnourished children [9]. In addition to increasing risk of many other childhood morbidities [10], the impacts of severe zinc deficiency include cognitive impairment [11,12], recurrent infections and diarrhea [10,13], and delayed wound healing [14]. In adolescents and adults, overt zinc deficiency impairs reproduction [15,16].

There is growing evidence that also links zinc deficiency with increased risk of cardiometabolic disorders [17]. Specifically, zinc plays an important role in insulin secretion and glucose homeostasis, and low zinc status has been associated with more severe type 2 diabetes mellitus [17,18]. Zinc status also affects lipid metabolism, including the absorption, synthesis, and metabolism of fatty acids, which impacts circulating lipid profiles and may increase risk of cardiovascular disease [18,19].

Progress toward preventing and combating zinc deficiency is stifled by the lack of sensitive and specific biomarkers for the assessment of individual zinc status [20,21]. Novel functional biomarkers, however, demonstrate potential in research studies and show promise for the monitoring of population-level interventions [20,22,23].

The purpose of this review is to explore recent developments in the understanding of zinc’s role in health and the prevalence of zinc deficiency, impacts of the changing global context on zinc intake, measurement of zinc status, and solutions for improving population zinc intake. The review underscores the necessity to implement evidence-based interventions to prevent and control zinc deficiency across the life course.

## The Roles of Zinc in Health: From Molecules to Mechanisms

The roles of zinc in critical health functions, including immunocompetence, are numerous and multifaceted. For example, T-cell activation (a key action in the immune response needed for the destruction of virus-infected cells and neoplasms [24]) requires the zinc-dependent formation of a complex between the protein Lck and the T-cell coreceptor CD4 or CD8 [25]. Zinc further regulates phosphorylation and dephosphorylation reactions in immune cell receptor signaling, and zinc finger structures are needed for transcriptional regulation of host defense genes [26]. Zinc is needed for the generation of acids and the concentration of toxic levels of zinc to kill pathogenic bacteria, for the catalytic activity of proteases that disassemble pathogen-derived proteins, and to catalyze the dismutation of cytotoxic superoxide anions generated in host defense [27].

The beneficial effects of therapeutic zinc supplementation have been observed in a variety of infections, including diarrhea and acute lower respiratory tract infections [24,28], 2 major causes of morbidity and death in children < 5 y in LMICs [29]. Preventive zinc supplementation in LMICs reduces the incidence of diarrhea, may decrease the incidence of acute lower respiratory tract infections, and reduces child mortality [[30], [31], [32]].

Zinc also has numerous critical roles in metabolic health. Zinc is needed in the formation of insulin crystals, a condition for their release into blood circulation [33]. Upon secretion with insulin, zinc signals pancreatic β-cells to prevent insulin over-release [34], and zinc-dependent regulation of phosphorylation increases insulin receptor sensitivity [35]. During lipolysis, albumin, the carrier protein for extracellular zinc, traffics free fatty acids from lipoprotein lipase to cells. Free fatty acids binding to albumin cause zinc release [36], which is taken up into tissues postprandially [37]. Albumin can become glycated with chronic exposure to elevated blood glucose, reducing its capacity to both carry zinc and to traffic free fatty acids [38,39]. Essential fatty acid metabolism, important in the regulation of inflammation, is also sensitive to changes in zinc intake [40]. Zinc is a key element in the response to oxidative stress in part through its catalytic role in superoxide dismutase [26] and signaling cellular response to oxidative stress upon release from metallothionein in the presence of reactive oxygen species [41]. Zinc further regulates vascular tone, induces vasorelaxation [42], and reduces the stiffness of clots [43].

It is through mechanisms such as these that subclinical zinc deficiency may precipitate a general dysregulation of metabolic function and inflammation that increases the burden of NCDs. Recent meta-analyses found that individuals with the highest dietary zinc intakes had a 13% lower risk of developing type 2 diabetes [44], and low-dose zinc supplementation significantly reduced risk factors for cardiovascular disease and type 2 diabetes, including high fasting blood glucose, insulin resistance, total cholesterol, and low-density lipoprotein cholesterol concentrations [45].

## The Burden of Zinc Deficiency

Zinc deficiency underlies all 3 coexisting burdens of malnutrition—undernutrition, micronutrient deficiencies, and overweight, obesity, and diet-related noncommunicable diseases [46,47]—and usually exists in combination with other micronutrient deficiencies.

To quantify the burden of health disparity attributable to zinc deficiency, the disability-adjusted life year (DALY) approach is useful to estimate the loss of life years due to disability or death. For example, a recent analysis considering the prevalence of inadequate zinc intake and disability or death due to stunting, diarrhea, and pneumonia estimated that 3.7 million DALYs were lost in China due to zinc deficiency in infants and children [48]. These estimates may underestimate the true burden; due to the broad functional importance of zinc throughout the life course, it is likely that DALYs attributed to other risk factors are also consequent—at least in part—to zinc deficiency.

DALYs lost due to NCDs are trending upward, whereas those lost due to infectious diseases continue to decrease [49]. Notably, elevated fasting plasma glucose, a key risk factor for multiple NCDs, ranked in the top 10 global DALY risk factors in 2010 [50]. Thus, a different picture of DALYs attributable to zinc deficiency emerges when the estimate is based on the contribution of zinc deficiency to elevated fasting blood glucose and the related NCDs [51]. Globally, per 100,000 population, 74.2 DALYs lost due to diabetes and kidney disease, 17.6 DALYs lost due to cardiovascular disease, and 8.8 DALYs lost due to cancer are attributable to elevated fasting plasma glucose from zinc deficiency. Due to zinc’s role in the regulation of glucose homeostasis, zinc deficiency is, thus, responsible for 7.1% of DALYs lost with these NCDs [51]. These data illustrate the importance of considering the multiple functional roles of zinc when estimating the burden of zinc deficiency.

## The Impact of Complex and Changing Environments on Dietary Zinc Intake

The ever-changing environments in which we live and in which our food grows impact our dietary zinc intake. A comprehensive modeling assessment of future socio-economic pathways and climate scenarios has projected that delivering micronutrient adequacy, including zinc, will remain a greater challenge than energy adequacy globally, especially for populations consuming lower amounts of zinc-rich foods [52].

Experiments with plants cultivated in growth chambers and under field conditions have shown that when crops are grown at elevated atmospheric CO_2_—at levels projected for 2050—there is a decline in the zinc concentrations in the grains of many staple cereal crops [53]. Although there are uncertainties about the combined effects of increased CO_2_ and increased temperatures, this carbon “nutrient penalty” has been projected to decrease the global availability of dietary zinc by 14.6% (and 13.6% for iron) by 2050 [54].

The performance of new biofortified genotypes (G) of crops bred to have increased grain nutrient concentration is influenced by the complex and changing environments (E) in which they are grown and the site-specific crop management (M) or agronomic approaches adopted by farmers. Together, these are known as G × E × M interactions. Many studies have evaluated and reported on the effects of applying zinc-containing fertilizers to cereal crops through foliar and soil applications to increase grain zinc concentrations—a process known as agronomic biofortification [55,56]. However, very few studies have attempted to quantify G × E × M interactions.

In a G × E × M field study on a biofortified wheat variety (Zincol-2016) in Pakistan, grain zinc concentration was reported to be greater than a local reference variety, but only under higher soil zinc concentrations [55]. At sites with low plant-available soil zinc concentrations, nutritional enhancement from plant breeding was not observed. In a recent E × M field study in Ethiopia [57], landscape position (e.g., elevation above sea level, precipitation, slope/drainage) influenced grain zinc concentration in wheat, which was linked to soil type. However, there were no interaction effects of landscape position and response to zinc fertilizers in either wheat or teff, which indicates that agronomic biofortification with zinc fertilizer is suitable across diverse field conditions.

A major challenge with G × E × M studies is achieving sufficient experimental power to detect small, albeit nutritionally impactful, potential effect sizes [55,56,58,59]. The use of locally sourced organic materials (as part of broader strategies to improve soil health and function) has been reported to moderately improve the zinc composition of grains in maize-based and wheat-based smallholder farming systems in Zimbabwe [60] and Ethiopia [61], respectively. This may be due to improved supply of other nutrients, such as nitrogen, that can augment zinc uptake and translocation from leaves to grains. In a comprehensive scoping review of the effect of “regenerative agriculture” practices—i.e., using agronomic methods designed to improve soil health—there were improvements in the grain zinc composition of rice in 15 out of 16 studies that reported on the impact of increased inputs of organic materials into soils [59].

Estimates of baseline zinc intakes are essential for forecasting the potential impacts of the changing environment and climate on nutritional status and estimating the impacts of interventions designed to help prevent and mitigate the effects of dietary inadequacies.

## Assessment of Human Zinc Status

There is a barrier to action that has inhibited progress in implementing interventions aimed at reducing zinc deficiency: the inability to measure their impact. Of the micronutrient deficiencies, that of zinc is the most insidious. Zinc deficiency and changes in zinc nutritional status often go undetected due to a lack of sensitive and specific zinc biomarkers. Although zinc nutriture clearly determines health, the breadth of zinc’s roles means that the signs of deficiency are difficult to relate to any specific biomarker. A clinical biomarker for zinc that, outside of a severe deficiency, can conclusively tell us when an individual is zinc deficient remains elusive [20].

The most frequently used biomarker of zinc status is plasma zinc concentration (PZC) [62]. However, monitoring PZC comes with several challenges. It fluctuates in an individual by 20% over the course of the day [63], and it is influenced by the length of overnight fast, the size and composition of the previous meal [63], and inflammation [64]. Even if these variables are controlled, analytical variability remains due to a lack of standardization and other factors [62]. Health-related effects of changes in zinc intake are also observable in controlled experimental settings before changes in PZC [[65], [66], [67]]. The over reliance on PZC thus inhibits our ability to identify functionally significant zinc deficiency or to monitor its resolution [62,64].

Despite the limitations of PZC, repeated, population-based measures are useful to estimate trends in zinc status. In Pakistan, for example, serial national and sub-national micronutrient surveys conducted over 20 y have demonstrated a significant reduction in the prevalence of zinc deficiency among children and females of reproductive age from 37% and 41% in 2001 to 18.6% and 22.1% in 2018, respectively [[68], [69], [70]].

Several functional biomarkers of zinc have been shown to be more sensitive than PZC to changes in zinc intake, and there is a trend toward their use in monitoring the efficacy and effectiveness of zinc interventions [23]. Examples include essential fatty acid desaturation, DNA damage, and zinc transporter gene expression [20,22,23,71]. Data from animal models further suggest that changes in gut microbiota may be used for the evaluation of zinc status [72]. Although demonstrated in research settings, the incorporation of such functional zinc biomarkers in the monitoring of community-based zinc interventions has been limited to date. Expanding the use of these measures in future zinc intervention studies is key to exposing subclinical zinc deficiency and curtailing its contribution to the triple burden of malnutrition.

Another approach that can be used to understand the prevalence of zinc deficiency among populations is the measurement of dietary zinc intake [20]. Yet, determining dietary zinc intakes is also difficult due to variations in data and data sources at national and sub-national levels.

On a national scale, dietary zinc intakes among populations can be inferred from zinc *supplies* using Food Balance Sheets (FBSs) produced by the Food and Agriculture Organization of the United Nations. FBS provide a proxy for the consumption of different food groups and are typically used to determine the prevalence of undernourishment based on the energy density of these food groups [73]. FBS data can be adapted to estimate per capita national level zinc supply by combining them with estimates of the zinc composition of different food groups and then comparing zinc supply with Estimated Average Requirements for zinc [4,5,74].

A major limitation of the FBS approach arises when using food composition data from single sources, especially for widely consumed staple crops, which assumes homogeneous access to food groups across socio-economic and demographic statuses. For example, using 2011 FBS and standard food composition data of produce from the United States, the global risk of zinc deficiency was estimated to be 16%, with ∼90% of this risk concentrated in Africa and Asia [4]. Across continental Africa, use of FBS and regional food composition tables have shown a 40% mean estimated risk of zinc deficiency, but with considerable variation between countries [74].

At sub-national scales, Household Consumption and Expenditure Surveys (HCES; surveys that record food consumption data at a household level) can be combined with local food composition data to estimate zinc intakes [75]. Access to adequate local food composition data is limited, however. In Malawi, HCES and zinc composition data based on local foods revealed that > 50% of the population is at risk of zinc deficiency due to inadequate intake [76].

Dietary zinc intakes also vary spatially, being lower in rural areas and associated with household socio-economic characteristics. For example, a study reported that 88% of households among the poorest wealth quintile had inadequate zinc supply to meet the sum of household members’ Estimated Average Requirements, compared to 28% in the wealthiest quintile [76]. Where food is grown and consumed locally or regionally, quantifying the spatial variation in the zinc composition of staple crops is an important consideration in understanding the prevalence of zinc deficiency.

Methods are being developed based on HCES to explore in more detail the potential of different zinc interventions among different socio-economic and other demographic groups and to explore the effects of seasonality of consumption of different food groups, which are often captured in HCES surveys [75].

Recent “GeoNutrition” surveys using nationally representative sampling designs showed substantial spatially correlated variation in grain zinc concentration in staple cereals at distances of over 100 km in both Ethiopia and Malawi [77]. This variation in grain zinc concentration arises due to soil (e.g., soil pH, soil organic matter content) and landscape properties, which are interlinked in complex ways [78]. These surveys are consistent with earlier studies, for example, in Malawi, that have shown the grain zinc concentration of maize grown on calcareous Vertisol soils was ∼30% higher than the grain zinc concentration from other typical soils, resulting in a greater dietary zinc intake based on direct analyses of composite diets (6.4 vs. 4.8 mg/d) [79]. Crop analysis and food intake data may thus provide an early warning of disproportionately high zinc deficiency risk, especially in localized areas practicing subsistence agriculture and can inform a need for more detailed surveys and potential zinc interventions.

There are strengths and limitations of each of the aforementioned methods to assess human zinc status (Table 1); whereas research must continue to improve these and other methods, addressing the global burden of zinc deficiency across the life course cannot wait. There is a clear and urgent need for the appropriate implementation of evidence-based interventions that are known to be efficacious, practical, and sustainable.

**TABLE 1:** Summary of strengths and limitations of the reviewed methods for assessing zinc status

Biomarker	Strengths	Limitations
**Biochemical**		
Plasma zinc concentration	Most widely used biomarker. Only biomarker marker that can be used to assess individual zinc status.	Results are easily confounded, e.g., by inflammation or fasting status. Careful control of confounding factors and analytical variation recommended; research on how to accomplish this is ongoing.
Novel functional biomarkers, e.g., fatty acid desaturation, DNA damage, zinc transporter gene expression, gut microbiome	Respond to changes in dietary zinc intake in research populations; have potential for greater sensitivity than plasma zinc concentration based on data from controlled studies.	Not specific to zinc. More evidence needed from community-based interventions.
**Dietary modeling**		
Dietary zinc intake using food balance sheets	Very low cost. FBS data readily available for 187 countries.	Assumes homogeneous access to food groups across socio-economic and demographic statuses. Food zinc composition is not geographically uniform.
Household Consumption and Expenditure Surveys and local food composition data	Zinc intake can be estimated for different socio-economic and demographic groups. Zinc composition of local food can be included.	Access to local food composition data is often limited. Intake survey data not always reliable.
Geospatial modeling of soil and crop zinc content	Enables predictive mapping of regions with disproportionately high zinc deficiency risk.	Limited data availability. Does not account for wider food system complexities (e.g., food transport, processing, etc.).

## Evidence-Based Interventions to Increase Population Zinc Intake

Increasing zinc intake and combating global zinc deficiency, particularly among the most vulnerable population groups, requires context-specific strategies that combine complementary evidence-based interventions where appropriate, including supplementation, large-scale food fortification (LSFF), and food and agricultural strategies such as soil zinc repletion strategies [80] and biofortification [81]. These strategies must be undertaken concurrently with efforts to improve zinc bioavailability and advance dietary diversification (the gold standard approach for achieving an optimal diet yet one that remains inaccessible for most of the global population) and alongside other multisectoral public health approaches that address the underlying causes of malnutrition and food insecurity.

Concurrent with the interventions discussed, it is essential to consider strategies to improve zinc bioavailability from food to enhance net zinc absorption and utilization [23]. Besides the total content of zinc, phytic acid (PA) present in plant-based foods is the most important dietary factor affecting zinc bioavailability. PA has a high affinity for divalent metals, including zinc, iron, and calcium, which bind PA phosphate groups to form insoluble and indigestible complexes in the gastrointestinal tract, thus reducing zinc absorption [82]. The amount of zinc and the ratio of phytate to zinc (PA:Zn) in the diet can determine the fraction of dietary zinc that is absorbed. The World Health Organization estimates that in an unrefined, vegetarian diet (such as those typical in LMICs), the PA:Zn molar ratio is > 15, and the fractional absorption of zinc from the diet is 15%. Whereas for a refined or mixed (animal- and vegetable-based) diet, the PA:Zn molar ratio is < 5, and the fraction of zinc absorbed is ≤50% [83]. Therefore, alongside increasing dietary zinc intake, it is important to reduce the PA:Zn molar ratio to improve zinc bioavailability without affecting other consumer preferences [84].

Supplemental zinc, commonly provided as a tablet or syrup, can be used as a targeted treatment strategy in response to an identified deficiency or prophylactically when high risk of deficiency has been identified. The chemical form of zinc used in supplements may be inorganic (e.g., sulfate, oxide, or citrate), organic (e.g., gluconate or malate), or zinc complexed to amino acids (e.g., histidine or lysine). The chemical form has an impact on the solubility and, thus, absorption of zinc from the gastrointestinal tract [85]. Whether the supplement is taken in the fasting state or with a meal also has an impact on how it is absorbed and metabolized [86,87]. A disadvantage of supplementation is that it requires behavior change; thus, the level of compliance has a major impact on its success [88].

Systematic reviews and meta-analyses of zinc supplementation (provided alone or with other micronutrients) in children and adults have demonstrated the effectiveness of this approach in increasing PZC in adults [89] and functional outcomes in children < 5 y old, including height, weight, and weight-for-age and weight-for-length [32,90]. Although some studies did not increase linear growth, they demonstrated impressive reductions in morbidity rates [[91], [92], [93]]. Zinc supplementation also reduces diarrhea-related morbidity [24,94] and incidence of fever and upper respiratory tract infections (92) but has no effect on pneumonia and malaria morbidity [[95], [96], [97]]. In addition, no significant impact on children’s behavioral or motor development has been observed [98]. There is no convincing evidence for the effectiveness of zinc supplementation on outcomes for pregnant females, except for a small effect on preterm births in LMICs [7,98,99].

An alternative means of supplementation to improve zinc status is the consumption of ready-to-use fortified foods. Fortification adds zinc to staple foods and/or common condiments during processing or directly to meals at home using multiple micronutrient powder sachets [100]. Fortification can be used to cost-effectively target populations through mass (or “universal”) fortification of foods or food products that are consumed regularly by a large proportion of the general population (e.g., cereal flours). Fortification can also target population subgroups using specific food vehicles and delivery mechanisms, for example, through complementary foods for young children, foods served within institutional programs for preschool or school-aged children, or foods delivered as part of an emergency response [101].

Several studies have examined the impact of zinc fortification on health outcomes, with mixed results. A systematic review of zinc fortification studies lasting 1–12 mo assessed health outcomes on females, infants (including preterm), and children ≤11 y old [102]. In these studies, staple foods, condiments, or processed foods were fortified exclusively with zinc. Pooled analysis revealed that zinc fortification was associated with a significant improvement in PZC and an increase in height velocity for newborns, but only within the subgroup with a very low birth weight. A more recent systematic review and meta-analysis of fortification of a range of food vehicles (such as grains, beverages, condiments, or combinations of >1 food item) with zinc in combination with other micronutrients evaluated outcomes on zinc-related biomarkers in males and females of all ages [103]. Pooled analysis revealed that multiple micronutrient fortification including zinc was associated with an increase in PZC, a reduced prevalence of zinc deficiency, and an increase in child weight (aged 1 – 14 y). A Cochrane review of the effect of zinc fortification of staple foods on the general population aged over 2 y old included 8 trials with a duration between 1 – 9 mo [6]. The pooled analysis revealed an increase in serum/PZC when zinc was added alone, although not when zinc was administered in combination with other micronutrients. Thus, some questions remain regarding the optimal composition and combination of nutrients added to fortified foods, and more research is needed to understand potential physiological interactions at the gastrointestinal and systemic levels.

Despite the prevalence of zinc deficiency in numerous LMICs and guidelines for zinc fortification [104], implementation of large-scale food fortification (LSFF) programs including zinc remains limited; as of 2021, only 29 of 72 mandatory national LSFF programs included zinc as a fortificant [105] and program coverage can exclude rural communities, who mainly depend on food grown themselves and do not have access to sources of fortified products.

Another promising approach to increasing zinc intake is through zinc biofortification, the enhancement of zinc content, and bioavailability of food crops [106]. Biofortification can be achieved through: 1) Conventional selective breeding, whereby existing seed or germplasm from food crops with naturally high nutrient density are identified and cross-bred to produce staple crops with desirable nutrition and agronomic traits; 2) Agronomic methods, which can be used in combination with conventional breeding, requiring the physical application of nutrients to the edible portion of crops or soil to improve the nutritional and health status of the crop [107,108]. In the case of zinc, this is achieved through application of zinc fertilizers to the soil, foliar spray, or both [55]; or 3) Genetic engineering methods, where the desirable genes are transferred between 2 unlike species (transgenesis), or between crossable species (cisgenesis), or by editing the genome of the crop of interest with more precise and site-specific (gene editing) methods. Products of gene editing may be considered cisgenic. Gene editing can reduce the time from discovery to commercialization of a crop variety by two-thirds, relative to conventional and transgenic plant breeding [106].

Transgenic techniques have been used to enhance the zinc content of rice and barley [109] and to improve the bioavailability of zinc from barley (through increased phytase activity by expression of the barley phytase gene HvPAPhy_a) [110]. Gene editing is currently being applied to > 40 crops around the world to tackle agronomic challenges affecting crop productivity; none of these projects address nutrient density or bioavailability [111]. Despite the potential advantages of genetic modification, it is currently not the method of choice for scaling up biofortification due to ethical, regulatory, ecological, and other concerns regarding the associated risks on human safety and planetary health [106,112]. In the example of barley, the phytase gene originated from the same or a naturally crossable species (through cisgenesis) and may go some way to alleviating some of these concerns [110]. To date, the potential for national and regional regulations to define products developed with gene editing as conventionally bred has been met with approval in some regions and strong disapproval in others [111].

Using conventional breeding techniques, zinc-enhanced varieties of wheat, rice, maize, pearl millet, and beans have been developed [113]. One of the challenges of using conventional breeding to biofortify crops with zinc is achieving maximum zinc content without concurrently increasing the PA:Zn molar ratio of the crop. A systematic review of 9 studies of the effectiveness of consuming zinc biofortified staple crops on zinc status revealed an increase in total zinc absorption among 5 studies associated with a reduction in the PA:Zn molar ratio, indicating enhanced zinc bioavailability [113]. Socio-economic studies have also assessed farmers’ and consumers’ evaluation of biofortified varieties and their willingness to adopt them with positive results, a prerequisite to scaling up [94,[114], [115], [116], [117]].

As drivers of food insecurity, such as conflict, climate change, and unfavorable socio-economic conditions, rise globally, it is vital that context-specific solutions to address malnutrition in all its forms (including micronutrient deficiencies) are prioritized [118]. Each approach to improving zinc intake has advantages and limitations depending on the population and context (Table 2) [88,119]. It is imperative to understand the baseline zinc status of the population and target zinc interventions in regions where zinc deficiency is prevalent, particularly in the first 1000 d of life when impacts on growth and development are largely irreversible.

**TABLE 2:** Advantages and disadvantages of global nutrition interventions [88,120]

Strategy	Advantages	Disadvantages
Zinc supplementation	Effective; Can be used for prevention and targeted treatment; Can be cost-effective.	Requires high and sustained coverage; Effectiveness limited to specific target groups (e.g., children 6–59 mo); Reliance on behavior change, poor compliance.
Zinc fortification of food	Effective with/without other micronutrients; Can be scaled; Can be used to target vulnerable groups; Minimal or no behavior change required.	Requires effective monitoring and quality control; Depending on setting, may not reach a large proportion of the population; Ongoing cost of zinc pre-mix; Additional cost passed on to consumers.
Zinc biofortification of staple crops	Effective; Can be scaled; Low cost once R&D of new variety complete; Can reach rural markets and farming communities; No behavior change required; Benefits the whole family.	Lengthy process using conventional breeding methods; Challenge to maximize bioavailability (breeding for zinc vs. phytic acid); Additional fertilizer cost if agronomic biofortification; GxE phenotypic expression variation.

## Discussion

Food insecurity and inadequate intake of dietary zinc are the main causes of suboptimal zinc status. The consequent biochemical and physiological sequelae described in this review contribute to economic and social impacts felt at the individual, community, and population levels. Invariably, it is the most vulnerable individuals and populations, including rural and urban poor females and children, that are affected by poor diets to the greatest extent.

There is ample evidence of the public health and socio-economic impact of zinc deficiency, as well as readily available and deployable solutions to prevent it. The proportion of DALYs lost to NCDs attributable to zinc deficiency emphasizes the critical importance of preventing and controlling zinc deficiency across the life course. However, the political will and global concerted action required to implement solutions effectively at scale must still be marshaled. Multiple platforms offered by current efforts to transform food and health care systems should be systematically seized to include preventive food-based and medicinal zinc interventions in ad hoc combinations for each context.

The pros and cons of supplementation, LSFF, and biofortification and their distinct characteristics reveal obvious complementarities for target population groups and markets. Preventive supplementation is not economically or logistically viable in most LMICs. This is demonstrated by the lack of progress in scaling the high impact World Health Organization and the United Nations Children’s Fund joint guideline for diarrhea management, which includes the addition of a short course of zinc supplementation [[120], [121], [122], [123]], and by the limited effectiveness of iron supplementation strategies in children and females of childbearing age [88,124,125]. Whereas large-scale fortification with zinc is a minimal risk and cost-effective approach for industrially produced staple foods and condiments, such commodities are often irregularly available or inaccessible to people living in extreme poverty, particularly in rural areas. Similar health benefits as those derived from fortified food consumption can be provided sustainably to those without access to these commodities by “preharvest” biofortification. This technology can enhance the zinc density in crops that are produced and consumed after minimal processing by rural farming communities (e.g., wheat, rice, maize, and pearl millet) as well as in crops that are not milled prior to cooking (e.g., common beans).

Inclusive nutrition strategies based on smallholder farming systems, such as zinc biofortification, are endorsed because they deliver essential micronutrients to large segments of the population without the need for behavior change [126]. When implemented alongside infectious disease control and social safety net programs, agricultural nutrition interventions can help transform food systems to produce diets that are inherently and sustainably more nutrient-dense and better equipped to offset climate-related shortfalls in food nutrient concentrations. Regular consumption of foods biofortified with zinc increases zinc absorption [113,[127], [128], [129]] and reduces maternal and childhood morbidity [130]. Zinc biofortification has had positive impacts on zinc biomarkers, and the potential to impact relevant health outcomes warrants further research, especially in programmatic settings.

Effective research communication of the agricultural, economic, and health benefits of biofortified crops must be further integrated into advocacy efforts directed at program managers and policymakers to support the complementary and cost-effective scale up of dietary diversification, LSFF, and biofortification—while avoiding unnecessary overlaps.

The impact of holistic national policies that tackle zinc deficiency and its underlying causes is measurable, albeit imperfectly. Improvements in child and maternal infectious morbidity and related disability and in adult-onset NCD await bold policy decisions.

## Author contributions

The authors’ responsibilities were as follows—NML, AGH, MRB, EB, and ZAB designed research; NML, AGH, MRB, JF, EB, and ZAB conducted research; NML, AGH, MRB, JF, EB, ZAB wrote the article; JF had primary responsibility for final content. All authors read and approved the final manuscript.

### Conflict of interest

The authors report no conflicts of interest.

### Funding

NML is supported by the Biotechnology and Biological Sciences Research Council of the United Kingdom (BBSRC) Global Challenges Research Fund, Grant Number BB/S013989/1. MRB is supported by the GeoNutrition project [INV-009129], funded by the Bill & Melinda Gates Foundation, and the Growing Health (BB/X010953/1) Institute Strategic Programme, funded by BBSRC. AGH, EB, and JF are supported by HarvestPlus (www.HarvestPlus.org), a global program working to develop and promote biofortified food crops that are rich in vitamins and minerals needed for good health. HarvestPlus’ principal donors are the UK government and the Bill & Melinda Gates Foundation. The views expressed do not necessarily reflect those of HarvestPlus.
